# Automated interstitial lung abnormalities detection at CT: external validation and potential recognition of traction bronchiectasis/bronchiolectasis

**DOI:** 10.1007/s11604-025-01917-z

**Published:** 2025-12-11

**Authors:** Yusei Nakamura, Taiki Fukuda, Kota Aoyagi, Masami Kawagishi, Yuki Ko, Noriaki Wada, Takuya Hino, Tomoyuki Hida, Meike W. Vernooij, Daniel Bos, Daan W. Loth, Masahiro Ozaki, Akihiro Koga, Heida Bjarnadottir, Valborg Gudmundsdottir, Gunnar Gudmundsson, Vilmundur Gudnason, Mizuki Nishino, David C. Christiani, Gary M. Hunninghake, Kousei Ishigami, Hiroto Hatabu

**Affiliations:** 1 Center for Pulmonary Functional Imaging, Department of Radiology, Brigham and Women’s Hospital and Harvard Medical School, 75 Francis Street, Boston, MA 02115 USA; 2 Department of Clinical Radiology, Graduate School of Medical Sciences, Kyushu University, Fukuoka, Japan; 3 Department of Radiology, The Jikei University School of Medicine, Tokyo, Japan; 4 Canon Medical Systems Corporation, Tochigi, Japan; 5 Canon Inc., Tokyo, Japan; 6 Department of Radiology & Nuclear Medicine, Erasmus MC, Rotterdam, The Netherlands; 7 Department of Epidemiology, Erasmus MC, Rotterdam, The Netherlands; 8 Department of Respiratory Medicine, Erasmus MC, Rotterdam, The Netherlands; 9 Department of Respiratory Medicine, Amphia, Breda, The Netherlands; 10 Institute for Advanced Diagnosis for Rare Diseases & Conditions K.K., Tokyo, Japan; 11 Faculty of Medicine, University of Iceland, Reykjavik, Iceland; 12 Icelandic Heart Association, Kopavogur, Iceland; 13 Department of Respiratory Medicine, Landspitali University Hospital, Reykjavik, Iceland; 14 Pulmonary and Critical Care Division, Department of Medicine, Massachusetts General Hospital and Harvard Medical School, Boston, MA USA; 15 Department of Environmental Health, Harvard TH Chan School of Public Health, Boston, MA USA; 16 Pulmonary and Critical Care Medicine, Brigham and Women’s Hospital and Harvard Medical School, Boston, MA USA

**Keywords:** CT, Interstitial lung abnormalities, Artificial intelligence, Traction bronchiectasis/bronchiolectasis, Machine learning, External validation

## Abstract

**Purpose:**

An artificial intelligence (AI) system for detecting interstitial lung abnormalities (ILA) was previously developed but requires external validation. This study aimed to examine the robustness across different populations and investigate associations between the system outputs and traction bronchiectasis/bronchiolectasis severity patterns.

**Materials and methods:**

CT scans from population-based samples of the Rotterdam Study (2018–2019) and the Age Gene/Environment Susceptibility Reykjavik (AGES-Reykjavik) Study (baseline CT: 2002–2006, follow-up CT: 2007–2011) were used in this secondary analysis of the two cohorts. The AI system calculated ILA probability score (AI score) in the range from 0 to 1. Three experienced readers evaluated independently all CT scans for ILA, and two chest radiologists assessed traction bronchiectasis/bronchiolectasis using the 4-scale traction bronchiectasis/bronchiolectasis index (TBI) for severity by consensus. Receiver operating characteristic (ROC) analysis and Kruskal–Wallis test were used for statistical analysis.

**Results:**

The system analyzed 932 CT scans of the Rotterdam Study (mean participant age, 79.6 years ± 4.3 (SD), 482 women) and 5242 CT scans of the AGES-Reykjavik Study (mean participant age, 76.4 years ± 5.6, 3032 women), and achieved area under the ROC curve of 0.841 (95% CI 0.804, 0.879) and 0.823 (95% CI 0.798, 0.847), respectively. AI scores correlated with readers’ certainty, decreasing from unanimous ILA cases to No-ILA cases. Higher baseline AI scores correlated with greater severity of traction bronchiectasis/bronchiolectasis (TBI-3: 0.931 [IQR, 0.911–0.932], TBI-2: 0.738 [IQR, 0.406–0.880], TBI-1: 0.537 [IQR, 0.317–0.761], TBI-0: 0.250 [IQR, 0.136–0.455]).

**Conclusion:**

The system demonstrated robust ILA detection performance across different populations, with AI scores showing associations with traction bronchiectasis/bronchiolectasis severity.

**Supplementary Information:**

The online version contains supplementary material available at 10.1007/s11604-025-01917-z.

## Introduction

Interstitial lung abnormalities (ILA) are incidental CT findings of non-dependent lung abnormalities, and these imaging findings include ground-glass opacities, reticular abnormalities, traction bronchiectasis/bronchiolectasis, honeycombing, and architectural distortion [1–3]. Recent studies have shown that ILA are associated with advanced age, tobacco smoking and other inhalational exposures, and genetic factors, and the presence of ILA has been found to be associated with worse clinical outcomes and higher mortality [1–10].

Currently, the standard for assessing ILA is subjective visual assessment, however there can be high inter-reader variability, and the visual reading is time intensive and requires expertise [11–13]. To address this issue, recent studies have utilized automated methods for detecting ILA [14–18]. As one of them, Hata et al. developed an automated probability-based detecting system for ILA based on artificial intelligence (AI) that generates probability score (AI score) ranging from 0 to 1, representing the likelihood of ILA presence on CT scans [19]. The system demonstrated good diagnostic accuracy in the Boston Lung Cancer Study, and validating these findings in additional cohorts with different patient demographics would be important. Furthermore, analyzing factors that influence the evaluation criteria of the AI system would provide insight into its evaluation process. When readers evaluate manually, traction bronchiectasis/bronchiolectasis and honeycombing are emphasized because they affect prognosis [20, 21]. Investigating how these established radiological features correlate with the AI score may reveal parts of the evaluation criteria of the method. In addition, recent studies have shown that traction bronchiectasis/bronchiolectasis progresses over time, leading to increased mortality [22, 23]. Evaluating whether the longitudinal changes in the AI score (ΔAI score) correlate with traction bronchiectasis/bronchiolectasis progression may provide preliminary insights into this relationship.

The primary objective of this study was to validate the automated ILA detection system across two distinctive population-based cohorts. Secondary analyses investigated correlations between AI scores and reader assessment patterns, including potential associations with traction bronchiectasis/bronchiolectasis severity.

## Materials and methods

### Study populations

This study was a secondary analysis of de-identified data from two cohorts: the Rotterdam Study and the Age Gene/Environment Susceptibility Reykjavik (AGES-Reykjavik) Study. Both studies were approved by their respective institutional review boards (Rotterdam: MEC 02.1015; AGES-Reykjavik: VSN 00–063) with written informed consent of all participants. The Rotterdam Study is a cohort of middle-aged and older residents of Rotterdam, Netherlands, and included 945 participants who underwent chest CT scans between May 2018 and December 2019, as part of a study on vascular calcification assessment [24, 25]. The AGES-Reykjavik Study, which involved people in Reykjavik born between 1907 and 1935, included 5496 baseline CT scans (2002–2006) and 3252 follow-up CT scans (2007–2011, approximately 5 years after baseline CT) [26]. Rotterdam Study had full lung coverage for the vascular calcification assessment protocols (carotid and mediastinal regions), while AGES-Reykjavik Study had insufficient coverage of the apical lung due to cardiovascular-focused imaging (cardiac and aortic regions).

**Fig. 1:**
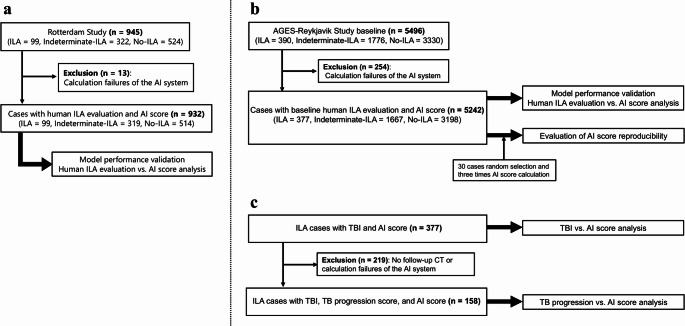
Inclusion and exclusion flowcharts. **a** Rotterdam Study. **b, c** AGES-Reykjavik Study. *AGES-Reykjavik Study* Age Gene/Environment Susceptibility Reykjavik Study, *AI* artificial intelligence, *ILA* interstitial lung abnormalities, *TB* traction bronchiectasis/bronchiolectasis, *TBI* traction bronchiectasis/bronchiolectasis index

CT acquisition parameters varied between cohorts. The Rotterdam Study used SIEMENS SOMATOM Drive scanners with 1.0 mm slice thickness and I30f or Br38f reconstruction kernels. The AGES-Reykjavik Study used SIEMENS Sensation 4 scanners with 2.5 mm slice thickness and predominantly B30f reconstruction kernels (with minority B30s). I30f, Br38f, and B30f represent soft tissue reconstruction kernels, and B30s represents a soft tissue kernel with slight edge enhancement. All participants in both cohorts (Rotterdam: 945, AGES-Reykjavik: 5496) received reader evaluation for ILA. AI analysis was successful in 932 of 945 (98.6%) Rotterdam Study cases and 5242 of 5496 (95.4%) AGES-Reykjavik Study cases. Analysis failures were primarily due to lung segmentation errors. Detailed inclusion and exclusion flowcharts are shown in Fig. 1.

### Automated ILA detecting system

The system is identical to that previously reported [19]. It employs a section inference model and a case inference model. The section inference model generates ILA probabilities for individual CT sections, and the case inference model integrates these to generate case-level probabilities using three classifiers. The system generates 12 different AI scores through a systematic combination of methodological approaches: (1) a section inference model using a two-label (ILA vs. No-ILA) or three-label (ILA vs. Indeterminate-ILA vs. No-ILA) classification method; (2) a case inference model similarly employing a two-label or three-label method; and (3) three types of classifiers (support vector machine [SVM], random forest [RF], and convolutional neural network [CNN]). This systematic approach generates 12 different models through all possible combinations (two section methods × two case methods × three classifiers = 12 combinations). Details are described in the original research [19].

To verify repeatability in the same CT scans, the system analyzed 30 randomly selected AGES-Reykjavik cases three times.

### ILA evaluation

In this study, ILA were defined based on the Fleischner Society criteria [1]. Chest CT scans were evaluated by up to three readers (thoracic radiologists [T.Hino, T.Hida, N.W., and H.H., with 7, 9, 10, and > 20 years of experience, respectively] and a pulmonologist [G.M.H., with > 20 years of experience]) using a sequential reading method [27]. The readers independently assessed CT scans as No-ILA, Indeterminate-ILA, or ILA [8, 9, 28]. Sequential reading involved hierarchical assessment: Reader 1 assessed all scans, Reader 2 assessed cases identified as ILA or Indeterminate-ILA by Reader 1 plus 20% of No-ILA cases, and Reader 3 resolved cases where disagreement occurred between Readers 1 and 2 [27]. Each reader was blinded to the prior assessment. The AI system successfully analyzed 932 cases from the Rotterdam Study and 5242 cases from the baseline AGES-Reykjavik Study, which were compared with visual assessments.

### Traction bronchiectasis/bronchiolectasis index (TBI)

TBI is assessed on a 4-point scale defined as 0, ILA without traction bronchiectasis/bronchiolectasis; 1, ILA with bronchiolectasis but without bronchiectasis or architectural distortion; 2, ILA with mild-to-moderate traction bronchiectasis; and 3, ILA with severe traction bronchiectasis and/or honeycombing [20]. Two thoracic radiologists (T.Hida and H.H, with 9 and > 20 years of experience, respectively) interpreted the AGES-Reykjavik Study CT scans and assessed TBI by consensus. A total of 377 cases were available for TBI versus AI score analysis from the baseline AGES-Reykjavik Study.

### Traction bronchiectasis/bronchiolectasis progression score

Two thoracic radiologists (T.Hino and H.H., with 7 and > 20 years of experience, respectively) compared baseline and follow-up ILA cases of the AGES-Reykjavik Study and assigned a traction bronchiectasis/bronchiolectasis progression score using a 5-point scale (1 = improved, 2 = probably improved, 3 = no change, 4 = probably progressed, 5 = progressed) [22] by consensus. For progression scoring, baseline and follow-up scans were reviewed side-by-side to assess temporal changes. Progressive change was defined as increasing traction bronchiectasis/bronchiolectasis severity or enlarging lung areas with traction bronchiectasis/bronchiolectasis. The “No-progression group” included participants with scores of 1, 2, or 3, and the “Progression group” included participants with scores of 4 or 5. The ΔAI score was defined as the difference between follow-up and baseline AI scores. A total of 158 cases were included in the traction bronchiectasis/bronchiolectasis progression versus AI score analysis.

### Reader agreement assessment

To test intrareader agreement and agreement with consensus assessment for ILA, TBI, and traction bronchiectasis/bronchiolectasis progression score, additional reading sessions were performed by a thoracic radiologist (Y.N., with 6 years of experience). To investigate agreement for ILA, the reader independently interpreted 50 and 300 randomly selected CT scans from the Rotterdam Study and the AGES-Reykjavik Study, respectively. In addition, to investigate agreement for TBI and traction bronchiectasis/bronchiolectasis progression score, 50 CT scans were randomly selected from 158 participants who were evaluated as ILA in both baseline and follow-up phases of the AGES-Reykjavik Study, and the reader independently interpreted these cases. After a 3-week interval, the reader conducted a second reading session, blinded to AI outputs, clinical information, and previous assessments. Intrareader agreement was calculated using two sessions by reader Y.N. Agreement with the consensus standard was evaluated between reader Y.N. and the consensus classification established by the other readers.

### Statistical analysis

Receiver operating characteristic (ROC) analysis was performed along with area under the receiver operating characteristic curve (AUC) calculation. Indeterminate-ILA cases were considered ILA negative in visual assessment for the primary analysis [29, 30]. A sensitivity analysis was conducted excluding Indeterminate-ILA cases to evaluate the potential impact of handling Indeterminate-ILA as No-ILA. Sensitivity and specificity were calculated with an AI score threshold of 0.5 and the Youden index. Continuous variables between two groups were compared using the Mann–Whitney U test. Continuous variables across three or more groups were compared using the Kruskal–Wallis test, followed by post hoc analysis using Dunn’s test with *P* values adjusted using the Benjamini–Hochberg method. Correlation between TBI and AI scores was assessed using Spearman’s rank correlation coefficient, with 95% confidence intervals estimated using bootstrap resampling with 1000 replicates.

Reader agreement was evaluated using the weighted kappa coefficient with quadratic weighting (κ_w_) and classified into the following categories: poor (0.00–0.20), fair (0.21–0.40), moderate (0.41–0.60), good (0.61–0.80), and excellent (0.81–1.00) [31].

Statistical analysis was performed using R (version 4.4.1), and statistical significance was defined as *p* < 0.05.

## Results

### Demographic characteristics and ILA evaluation

Of the 945 Rotterdam Study cases, 13 cases were excluded due to the AI system calculation failures. Among the remaining 932 cases (mean participant age, 79.6 years ± 4.3 [SD]; 482 women [51.7%]), 99 (10.6%) were assessed by readers as ILA, 319 (34.2%) as Indeterminate-ILA, and 514 (55.2%) as No-ILA. In the AGES-Reykjavik Study, 254 of 5496 cases were excluded due to the AI system calculation failures. Of the remaining 5242 cases (mean participant age, 76.4 years ± 5.6 [SD]; 3032 women [57.9%]), 377 (7.2%) were assessed by readers as ILA, 1667 (31.8%) as Indeterminate-ILA, and 3198 (61.0%) as No-ILA. The demographic characteristics were summarized in Table 1.

**Table 1:** Participant demographic characteristics

Characteristic	Rotterdam study (n = 932)	AGES-Reykjavik study (n = 5242)
Mean age (y)^*^	79.6 ± 4.3	76.4 ± 5.6
*Sex*		
Female	482 (51.7)	3032 (57.9)
Male	450 (48.3)	2207 (42.1)
Unknown^†^	0	3
*Smoking status*		
Former	549 (58.9)	2262 (44.6)
Current	47 (5.0)	628 (12.4)
Never	336 (36.1)	2179 (43.0)
Unknown^†^	0	173
Mean smoking pack-years^*^	12.7 ± 18.4	12.3 ± 19.5
Unknown	2	173

Categorical data are presented as numbers of participants, with percentages in parentheses

*AGES-Reykjavik Study* Age Gene/Environment Susceptibility Reykjavik Study

^†^ Not included in calculation of percentages

^*^ Continuous data are presented as means ± SDs

### Model performance

**Fig. 2:**
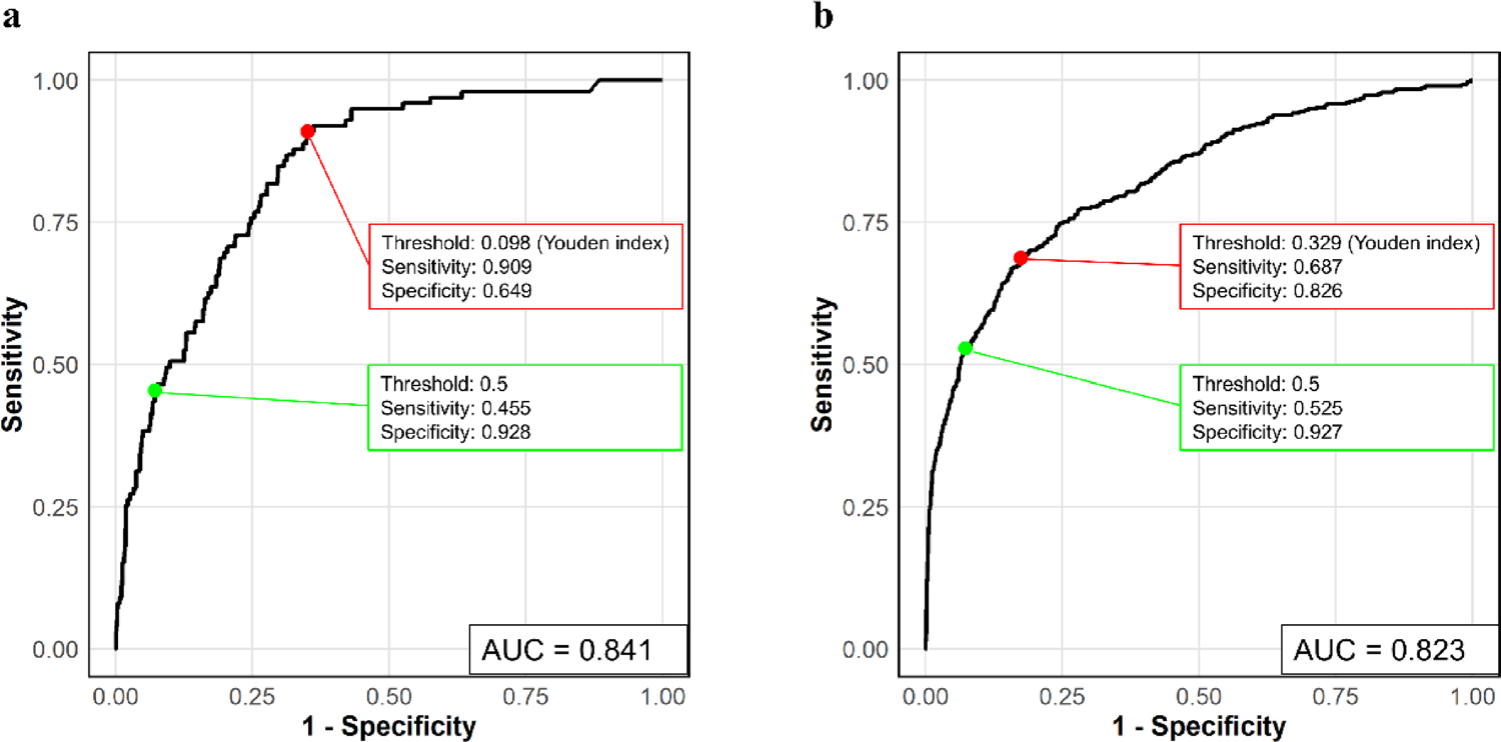
Receiver operating characteristic curves for the optimal models in the two cohorts. **a** In the Rotterdam Study, the highest AUC of 0.841 was achieved using the two-label method for the section inference model and the two-label method and random forest classifier for the case inference model. The sensitivity and specificity at a threshold of 0.5 and at the threshold of Youden index are shown in the figure (green and red points, respectively). **b** In the AGES-Reykjavik Study, the highest AUC of 0.823 was achieved using the three-label method for the section inference model and the three-label method and support vector machine classifier for the case inference model. The sensitivity and specificity are shown in the figure similarly. *AGES-Reykjavik Study* Age Gene/Environment Susceptibility Reykjavik Study, *AUC* area under the receiver operating characteristic curve

In the Rotterdam Study, the highest performance (AUC = 0.841; 95% CI 0.804, 0.879) was achieved by the pre-trained RF classifier using the two-label method in both slice inference and case inference models. Using a threshold of 0.5, sensitivity and specificity were 0.455 and 0.928, respectively. At the Youden index threshold of 0.098, sensitivity and specificity were 0.909 and 0.649 (Table 2). In the AGES-Reykjavik Study, the highest performance (AUC = 0.823; 95% CI 0.798, 0.847) was achieved by the pre-trained SVM classifier using the three-label method in both slice inference and case inference models. At the 0.5 threshold, sensitivity and specificity were 0.525 and 0.927, while at the Youden index threshold of 0.329, they were 0.687 and 0.826 (Table 2). The ROC curves of the best-performing model are shown in Fig. 2, and all 12 models’ results are summarized in Tables S1 and S2. Cross-cohort validation showed that the Rotterdam optimal model achieved AUC of 0.813 in AGES-Reykjavik Study and the AGES-Reykjavik optimal model achieved AUC of 0.838 in Rotterdam Study (detailed results in Tables S1 and S2). For the subsequent analyses presented below, we used the AI scores of the model that achieved the highest AUC in each cohort.

**Table 2:** Performance measures of optimal models

Study	Section inference method, Case inference method, and Case inference classifier	AUC	Threshold = 0.5		Threshold = Youden Index		Youden Index
			Sensitivity	Specificity	Sensitivity	Specificity	
Rotterdam study	Two-label section inference, Two-label case inference, and RF classifier	0.841 (0.804, 0.879)	0.455 (0.354, 0.545); 45/99	0.928 (0.910, 0.945); 773/833	0.909 (0.778, 0.960); 90/99	0.649 (0.615, 0.791); 541/833	0.098
AGES-Reykjavik study	Three-label section inference, Three-label case inference, and SVM classifier	0.823 (0.798, 0.847)	0.525 (0.475, 0.576); 198/377	0.927 (0.920, 0.935); 4512/4865	0.687 (0.637, 0.780); 259/377	0.826 (0.739, 0.864); 4018/4865	0.329

Data in parentheses are 95% CIs. Proportions are numbers of participants

*AGES-Reykjavik Study* Age Gene/Environment Susceptibility Reykjavik Study, *AUC* area under the receiver operating characteristic curve, *RF* random forest, *SVM* support vector machine

To evaluate the potential impact of classifying Indeterminate-ILA cases as ILA negative in the primary analysis, we conducted a sensitivity analysis limited to clear classification (ILA vs. No-ILA). When analyzing only ILA (n = 99) and No-ILA (n = 514) cases in the Rotterdam Study, the optimal model achieved an AUC of 0.894 (95% CI 0.861, 0.927). Using a threshold of 0.5, sensitivity and specificity were 0.455 and 0.969, respectively. At the Youden index threshold of 0.092, they were 0.919 and 0.759. In the AGES-Reykjavik Study, when analyzing ILA (n = 377) and No-ILA (n = 3198) cases, the optimal model showed an AUC of 0.864 (95% CI 0.841, 0.886). At the 0.5 threshold, sensitivity and specificity were 0.525 and 0.974, while at the Youden index threshold of 0.329, they were 0.687 and 0.898. The ROC curves for sensitivity analysis are presented in Figs. S1 and S2, and the performance indices are shown in Table S3.

### ILA visual assessment versus AI score analysis

**Fig. 3:**
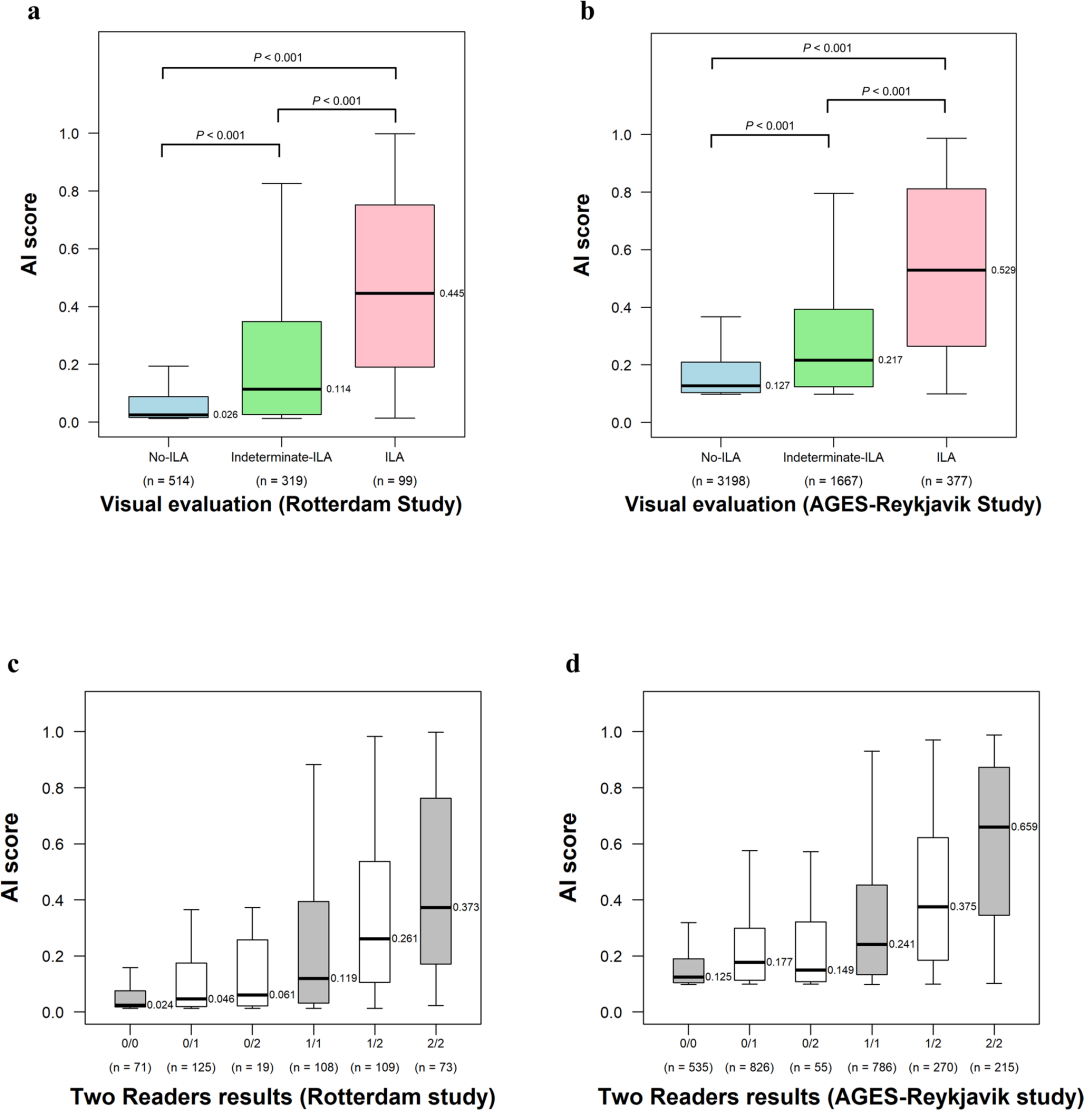
Box plots show the relationship between visual ILA evaluation and AI score. The y-axis represents the AI score. **a, b** The x-axis shows the final visual ILA evaluation determined by the three readers. Median AI scores were highest for ILA, followed by Indeterminate-ILA, and lowest for No-ILA in both cohorts. **c, d** The x-axis shows the results from Reader 1 and Reader 2, where 0, 1, and 2 represent No-ILA, Indeterminate-ILA, and ILA, respectively. 0/0, 1/1, and 2/2 indicate the cases unanimously classified as No-ILA, Indeterminate-ILA, and ILA by both readers, respectively. While 0/1, 0/2, and 1/2 indicate the cases with reader disagreement, for instance, 0/1 indicates one reader classified it as No-ILA while the other as Indeterminate-ILA. No significant differences were observed between 0/1 vs. 0/2 (*p* = 0.22) and 0/2 vs. 1/1 (*p* = 0.19) in the Rotterdam Study, and 0/1 vs. 0/2 (*p* = 0.32) in the AGES-Reykjavik Study. All other inter-group comparisons showed statistically significant differences. The details of *p* values are shown in Table S3 and S4. In both cohorts, median AI scores showed an ascending trend that correlated with the readers’ tendency to assess cases as ILA. *AGES-Reykjavik Study* Age Gene/Environment Susceptibility Reykjavik Study, *AI* artificial intelligence, *ILA* interstitial lung abnormalities

The median AI scores were significantly different across ILA categories in both cohorts (both *p* < 0.001): (0.445 [IQR, 0.190–0.752], 0.114 [IQR, 0.026–0.348], and 0.026 [IQR, 0.016–0.088] for ILA, Indeterminate-ILA, and No-ILA, respectively) in the Rotterdam Study and (0.529 [IQR, 0.265–0.811], 0.217 [IQR, 0.125–0.393], and 0.127 [IQR, 0.104–0.209], respectively) in the AGES-Reykjavik Study (Fig. 3a and b).

When comparing evaluations by Reader 1 and Reader 2 with AI scores, both cohorts showed similar patterns (Fig. 3c and d). Cases unanimously classified as ILA by both readers demonstrated the highest median AI scores (0.373 [IQR, 0.171–0.763] in the Rotterdam Study; 0.659 [IQR, 0.345–0.872] in the AGES-Reykjavik Study), while those unanimously classified as No-ILA showed the lowest scores (0.024 [IQR, 0.018–0.075] and 0.125 [IQR, 0.104–0.190], respectively). In both cohorts, cases with Indeterminate-ILA or reader disagreement showed AI scores that correlated with the perceived likelihood of ILA presence. Specifically, a descending trend in AI scores was observed across the following groups: cases with reader disagreement between ILA and Indeterminate-ILA (0.261 [IQR, 0.106–0.536] in the Rotterdam Study; 0.375 [IQR, 0.185–0.619] in the AGES-Reykjavik Study), cases unanimously classified as Indeterminate-ILA (0.119 [IQR, 0.032–0.392] and 0.241 [IQR, 0.133–0.453], respectively), and cases with reader disagreement between Indeterminate-ILA and No-ILA (0.046 [IQR, 0.019–0.174] and 0.177 [IQR, 0.114–0.298], respectively). The *p* values among these groups were shown in Fig. 3, Tables S4 and S5.

### AI score repeatability

The AI system analyzed 30 randomly selected AGES-Reykjavik cases three times, producing identical AI scores to the fifth decimal place across all models in all trials (Tables S6).

### TBI and traction bronchiectasis/bronchiolectasis progression versus AI score analysis

**Fig. 4:**
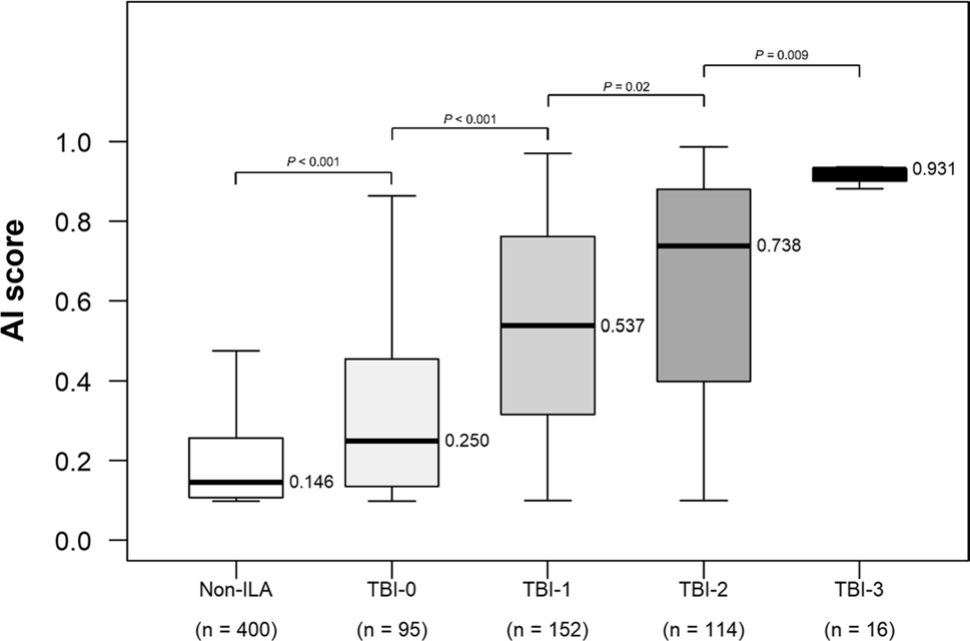
Box plots show the relationship between TBI and AI score. To address potential selection bias, 400 stratified random samples from Non-ILA cases (combining No-ILA and Indeterminate-ILA) were included as reference. The y-axis represents AI score. Higher TBI was associated with higher median AI score. Adjacent group comparisons showed statistically significant differences: Non-ILA vs. TBI-0 (*p* < 0.001), TBI-0 vs. TBI-1 (*p* < 0.001), TBI-1 vs. TBI-2 (*p* = 0.02), and TBI-2 vs. TBI-3 (*p* = 0.009). *AI* artificial intelligence, *ILA* interstitial lung abnormalities, *TBI* traction bronchiectasis/bronchiolectasis index

To address potential selection bias, we included 400 stratified random samples from Non-ILA cases (combining No-ILA and Indeterminate-ILA) as reference. Among all groups, median AI scores differed significantly across categories: 0.146 (IQR, 0.107–0.257) for Non-ILA, 0.250 (IQR, 0.136–0.455), 0.537 (IQR, 0.317–0.761), 0.738 (IQR, 0.406–0.880), and 0.931 (IQR, 0.911–0.932) for TBI-0, 1, 2, and 3, respectively (Fig. 4). Adjacent group comparisons were as follows: Non-ILA vs. TBI-0 (*p* < 0.001), TBI-0 vs. TBI-1 (*p* < 0.001), TBI-1 vs. TBI-2 (*p* = 0.02), and TBI-2 vs. TBI-3 (*p* = 0.009). All comparison *p*-values are shown in Table 3. Among ILA cases, the AI score showed a moderate positive correlation with TBI severity (Spearman’s ρ = 0.491; 95% CI 0.399, 0.576, *p* < 0.001).

**Table 3:** Relationship between TBI and AI score

Group	n	AI score^*^	*P* Value			
			vs. Non-ILA	vs. TBI-0	vs. TBI-1	vs. TBI-2
TBI-3	16 (4)	0.931 (0.911–0.932)	*p* < 0.001^‡^	*p* < 0.001^‡^	*p* < 0.001^‡^	*p* = 0.009^‡^
TBI-2	114 (30)	0.738 (0.406–0.880)	*p* < 0.001^‡^	*p* < 0.001^‡^	*p* = 0.02^‡^	–
TBI-1	152 (40)	0.537 (0.317–0.761)	*p* < 0.001^‡^	*p* < 0.001^‡^	–	
TBI-0	95 (25)	0.250 (0.136–0.455)	*p* < 0.001^‡^	–		
Non-ILA^†^ (Reference)	400	0.146 (0.107–0.257)	–			

Data are numbers of participants, with percentages in parentheses for TBI groups only (percentages calculated among ILA cases, n = 377)

*AI* artificial intelligence, *ILA* interstitial lung abnormalities, *TBI* traction bronchiectasis/bronchiolectasis index

^*^ AI score is used to refer to the ILA probability score calculated by the system in this study. Data are medians, with IQRs in brackets

^†^Non-ILA represents 400 stratified random samples from Non-ILA cases (No-ILA and Indeterminate-ILA combined) included as reference

^‡^ *P*-values were calculated using Kruskal–Wallis test followed by Dunn’s post hoc test with Benjamini–Hochberg correction

**Fig. 5:**
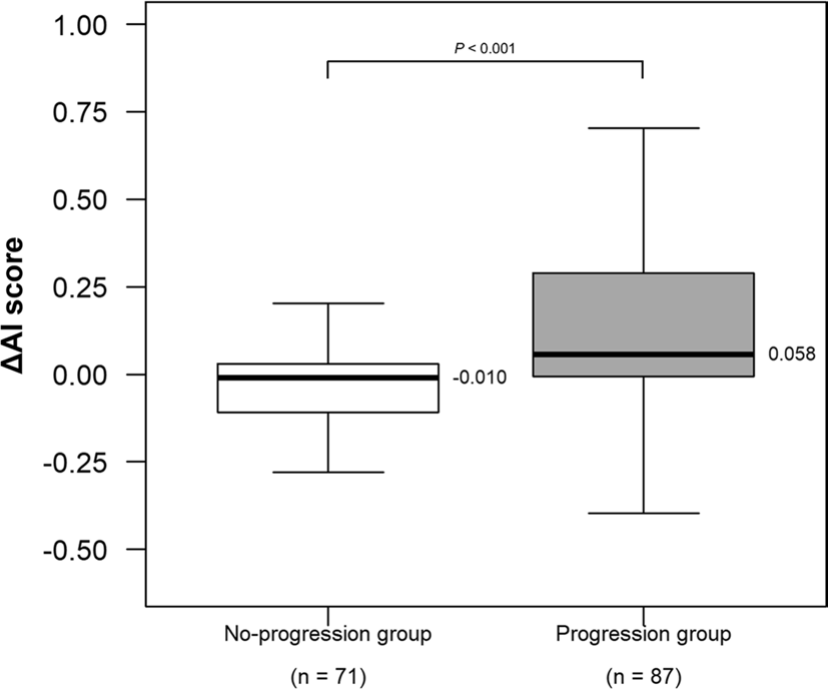
Box plots show the relationship between traction bronchiectasis/bronchiolectasis progression and ΔAI score. The ΔAI score was defined as the difference between follow-up and baseline AI score. The Progression group exhibited significantly higher median ΔAI score compared to the No-progression group (*p* < 0.001). *AI* artificial intelligence

**Fig. 6:**
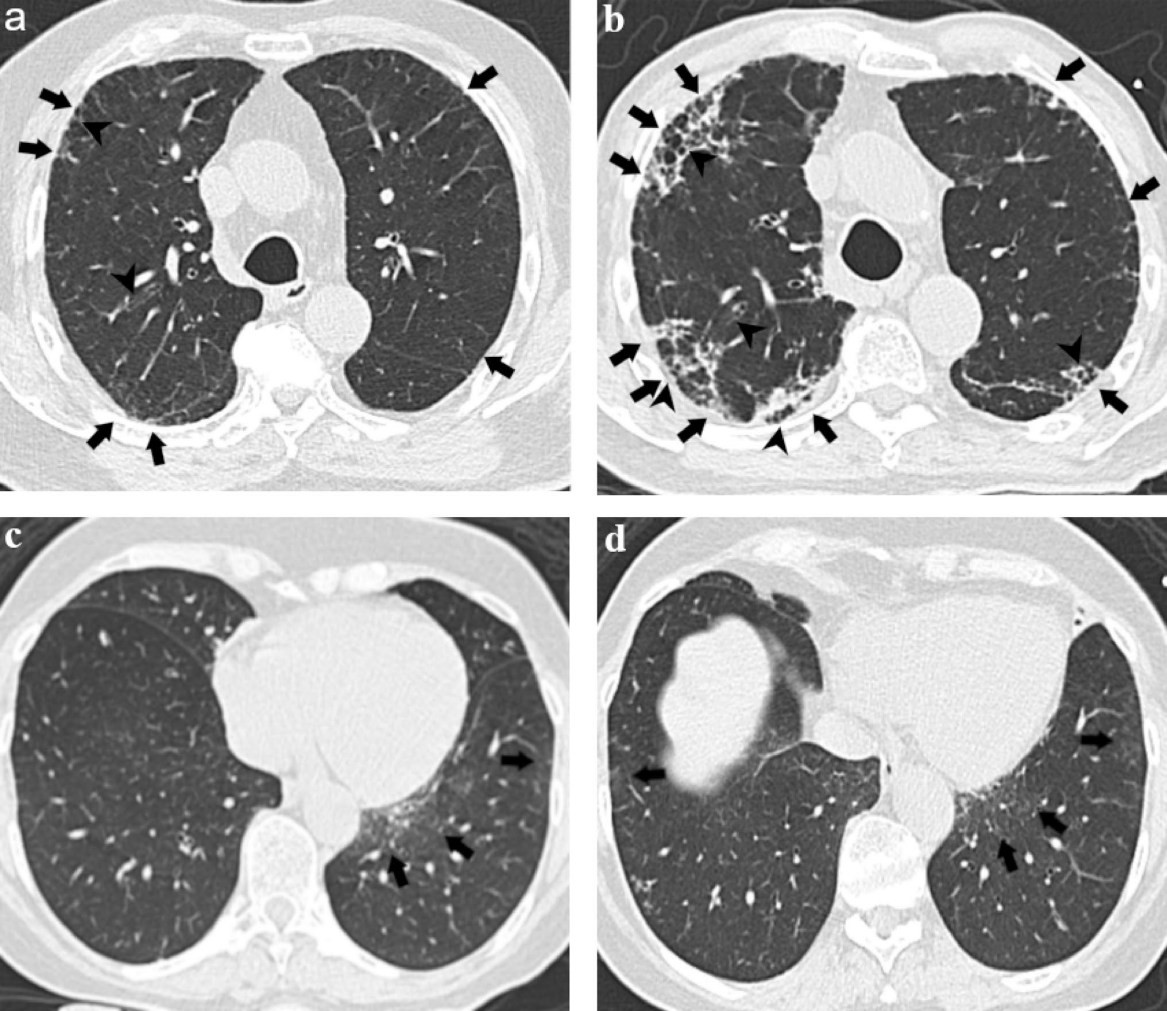
Representative cases. **a** and **b** show baseline and follow-up CT images of a patient, while **c** and **d** show those of another participant. **a, b** A participant assessed as Progression group by the readers (TBI-2 at baseline, TBI-3 at follow-up, and traction bronchiectasis/bronchiolectasis progression score = 5). The ground-glass opacities (arrows in **a**) and mild-to-moderate traction bronchiectasis (arrowheads in **a**) observed at baseline worsened during follow-up, showing progression to reticular patterns (arrows in **b**) and severe traction bronchiectasis (arrowheads in **b**). The AI scores were 0.426 at baseline and 0.934 at follow-up, with ΔAI score of 0.508. **c, d** A participant assessed as No-progression group by the readers (TBI-0 at baseline, TBI-0 at follow-up, and traction bronchiectasis/bronchiolectasis progression score = 3). Ground-glass opacities are observed in the subpleural regions of the lungs (arrows in **c** and **d**), without traction bronchiolectasis. The AI scores were 0.349 at baseline and 0.375 at follow-up, with ΔAI score of 0.026. *AI* artificial intelligence, *TBI* traction bronchiectasis/bronchiolectasis index

Among 158 AGES-Reykjavik ILA cases which had available follow-up scans, median ΔAI score differed significantly between Progression and No-progression groups (0.058 [IQR, -0.007–0.289] vs. -0.010 [IQR, -0.109–0.030], *p* < 0.001) (Table 4, Fig. 5). ROC analysis for ΔAI score discrimination of traction bronchiectasis/bronchiolectasis progression showed an AUC of 0.710 (95% CI 0.630, 0.791) (Fig. S3). At the Youden index threshold of 0.039, sensitivity and specificity were 0.574 and 0.775, with positive predictive value of 0.758 and negative predictive value (NPV) of 0.598. The NPV of 0.598 indicates that more than 40% of cases classified as No-progression group would actually be progressing. Representative cases are shown in Fig. 6.

**Table 4:** Relationship between traction bronchiectasis/Bronchiolectasis progression and ΔAI score

Traction bronchiectasis/bronchiolectasis progression score	n = 158	ΔAI score^*^	*P* Value			
			vs. score 1	vs. score 2	vs. score 3	vs. score 4
Score 5	56 (35)	0.048 (− 0.003 to 0.320)	*p* = 0.004^§^	*p* = 0.03^§^	*p* = 0.001^§^	*p* = 0.40^§^
Score 4	31 (20)	0.073 (− 0.013 to 0.217)	*p* = 0.006^§^	*p* = 0.04^§^	*p* = 0.008^§^	–
Score 3	65 (41)	− 0.008 (− 0.102 to 0.038)	*p* = 0.04^§^	*p* = 0.14^§^	–	
Score 2	2 (1)	− 0.121 (− 0.149 to − 0.092)	*p* = 0.46^§^	–		
Score 1	4 (3)	− 0.276 (− 0.338 to − 0.204)	–			
Progression group^†^	87 (55)	0.058 (− 0.007 to 0.289)	*p* < 0.001¶			
No-progression group^‡^	71 (45)	− 0.010 (− 0.109 to 0.030)				

Data are numbers of participants, with percentages in parentheses

*AI* artificial intelligence, *ILA* interstitial lung abnormalities

^*^ ΔAI score is defined as the difference between follow-up and baseline AI score. Data are medians, with IQRs in brackets

^†^ Progression group = traction bronchiectasis/bronchiolectasis progression score 4 or 5

^‡^ No-progression group = traction bronchiectasis/bronchiolectasis progression score 1, 2, or 3

^§^ *P*-values for comparisons among traction bronchiectasis/bronchiolectasis progression score categories (score 1–5) were calculated using Kruskal–Wallis test followed by Dunn’s post hoc test with Benjamini–Hochberg correction

^¶^ *P*-value for comparison between Progression group and No-progression group was calculated using Mann–Whitney U test

### Reader agreement assessment

Intrareader agreements of ILA were good in both cohorts (κ_w_ = 0.766; 95% CI 0.616, 0.917 in the Rotterdam Study and κ_w_ = 0.753; 95% CI 0.690, 0.816 in the AGES-Reykjavik Study). Intrareader agreement of TBI was good (κ_w_ = 0.780, 95% CI 0.608, 0.953) and that of traction bronchiectasis/bronchiolectasis progression score was excellent (κ_w_ = 0.869, 95% CI 0.791, 0.947).

Agreement with consensus standard of ILA was moderate in both cohorts (κ_w_ = 0.498, 95% CI 0.256, 0.740 in the Rotterdam Study and κ_w_ = 0.581, 95% CI 0.482, 0.680 in the AGES-Reykjavik Study). Agreement with consensus standard of TBI was good (κ_w_ = 0.745, 95% CI 0.629, 0.862) and that of traction bronchiectasis/bronchiolectasis progression score was also good (κ_w_ = 0.776, 95% CI 0.648, 0.903).

## Discussion

In this study, we externally validated the automated ILA detecting method previously reported by Hata et al. Then, we investigated the repeatability of AI scores, the system’s evaluation characteristics, and the association with TBI and traction bronchiectasis/bronchiolectasis progression. The system was applied to chest CT scans from the population-based samples of the Rotterdam Study and the AGES-Reykjavik Study, achieving model AUCs of 0.841 (95% CI 0.804, 0.879) and 0.823 (95% CI 0.798, 0.847), respectively. It was demonstrated that the system generates identical AI score for the same CT scan. The scores were correlated with ILA assessment by readers, with highest scores for cases unanimously classified as ILA and lowest scores for cases unanimously classified as No-ILA, and a gradually declining trend for cases where readers had different assessments. Furthermore, a positive correlation was found between TBI and AI scores.

In the Rotterdam Study and the AGES-Reykjavik Study, the AI system performed equivalent to the previous study using the Boston Lung Cancer Study (AUC 0.87) [19], and it suggests the system’s robustness across different populations (e.g. high risk screening versus general population) and different settings (different scanners and protocols). To assess the potential impact of classifying indeterminate ILA cases as negative, we performed a sensitivity analysis excluding these cases. This additional analysis demonstrated a higher AUC (Rotterdam: 0.894, AGES-Reykjavik: 0.864) compared with the primary analysis (Rotterdam: 0.841, AGES-Reykjavik: 0.823). This result suggests that the primary analysis does not overestimate performance. Rather, this performance difference reflects the difficulty of diagnosing Indeterminate-ILA cases, which remains challenging even for experienced readers. Importantly, this AI system maintained robust performance (AUC > 0.82) even when those difficult cases were included, demonstrating its practicality in clinical settings where such borderline cases would present frequently. In addition, we also evaluated robustness of various pre-trained methods and found that the deep-learning CNN-based model did not perform better than traditional machine learning approaches (SVM and RF). This result was the same as the previous study [19], and this is possibly due to the limited sample size available for training.

An important finding of this study is the distinction between model robustness and threshold generalizability. Cross-cohort validation showed robust discriminative performance with minimal AUC change (Rotterdam Study using AGES-Reykjavik optimal model: 0.838 vs. AGES-Reykjavik Study using Rotterdam optimal model: 0.813). However, there was substantial variation in the optimal threshold (Rotterdam Study: 0.098 vs. AGES-Reykjavik Study: 0.329). This primarily reflects differences in the optimal model for each cohort (Rotterdam Study: two-label section inference, two-label case inference and RF classifier vs. AGES-Reykjavik Study: three-label section inference, three-label case inference and SVM classifier). Importantly, there was still large variability in the threshold even when the same model was used across both cohorts. The model with three-label section inference, three-label case inference and SVM classifier showed a difference of approximately two times (Rotterdam Study: 0.169 vs. AGES-Reykjavik Study: 0.329), while the model with two-label section inference, two-label case inference and RF classifier yielded a difference of approximately three times (Rotterdam Study: 0.098 vs. AGES-Reykjavik Study: 0.283). This threshold variation suggests that factors other than model architecture, such as imaging parameters (e.g., slice thickness and reconstruction kernels), scanner characteristics, and population-specific factors (e.g., ILA prevalence and demographics), affect the optimal threshold. Although this AI system has robust ability to distinguish ILA from No-ILA, these factors may shift the score distribution. Consequently, site-specific threshold calibration is crucial before clinical application.

In this study, we demonstrated that this AI system produces repeatable results when analyzing the same CT images. It is important to verify the repeatability of their assessments before introducing AI-based imaging diagnostic systems into clinical practice. This is because if an AI system produces inconsistent assessments for the same images across different analyses, the reliability of its assessments will be compromised, potentially leading to inappropriate diagnoses and therapeutic interventions.

This study showed a correlation between the AI score and ILA visual assessment, and it indicates that the AI system may capture the subtle CT imaging difference like radiologists when assessing ILA. Furthermore, a correlation between the AI score and TBI was also demonstrated. This suggests that AI scores may correlate with imaging features associated with traction bronchiectasis/bronchiolectasis severity. Given that traction bronchiectasis/bronchiolectasis is emphasized in visual ILA assessment due to its prognostic significance, this correlation suggests that the system responds to imaging patterns that are also considered important by radiologists.

To evaluate potential associations between AI scores and morphological progression, we investigated the relationship between traction bronchiectasis/bronchiolectasis progression score and ΔAI score. At the group level, ΔAI score was significantly higher in the Progression group compared with the No-progression group. However, ROC analysis revealed important limitations for its application to individual participants. The AUC of 0.710 showed moderate performance, but the NPV of 0.598 demonstrated that this method is clinically unreliable for ruling out progression. It means that more than 40% of cases classified as No-progression group by this AI system actually progressed, which is clinically unacceptable. Multiple factors may contribute to this limited performance. The substantial overlap in distributions between groups suggests that the ΔAI score cannot reliably distinguish individual cases. Furthermore, a ceiling effect in participants with high baseline AI scores may limit the detection of progression. Because AI scores range from 0 to 1, participants with high baseline scores have limited room for score increase even if they actually have traction bronchiectasis/bronchiolectasis progression. Therefore, although this analysis demonstrates a statistically significant association between groups, its performance is currently insufficient for clinical decision-making in individual participants, and the ΔAI score should not be used for progression monitoring in clinical practice.

This study has several limitations. First, although visual assessment was used as the reference standard, interreader agreement was only moderate (κ_w_ = 0.498–0.581), meaning the inherent instability of the reference standard itself. This is not simply a study limitation, but reflects the fundamental challenges of diagnosing ILA, a subtle interstitial abnormality. This agreement is consistent with previously reported values for ILA assessment [11–13], highlighting the limitations of establishing a reference standard for AI validation in this field. This noisy reference standard, which differs among experienced readers, sets a theoretical upper limit for the performance of automated systems. Given moderate interreader agreement for the reference standard, perfect ILA identification using automated detecting system is theoretically difficult. In this context, the AUC of 0.82–0.84 for this AI system suggests a high level of performance within an inherently ambiguous task and approaches the upper limit imposed by expert disagreement. In addition, the lack of clinical outcomes in this study further limits the possibility of establishing a true gold standard. Neither visual nor AI assessment is absolute, and detailed case-by-case analysis comparing discordant cases may provide valuable insights. Second, in this study, we selected the optimal model based on AUC performance and determined the threshold using the Youden index. However, the optimal model and threshold should ultimately be determined based on clinical outcomes (e.g., mortality) and clinical application purposes (e.g., screening and triage). Sensitivity should be prioritized for screening purposes, while a high threshold that emphasizes specificity may be appropriate for triage. The substantial difference in optimal thresholds between cohorts may reflect differences in both optimal models and imaging parameters. A validating study based on clinical outcomes is currently underway to determine the optimal model and threshold. Third, the large temporal gap between cohorts (AGES-Reykjavik: 2002–2011 vs. Rotterdam: 2018–2019) represented a potential source of domain shift effects due to evolving CT technology, scanner differences, and protocol variations. In addition, the AGES-Reykjavik Study had incomplete coverage of the lung apex and used a thicker slice thickness (2.5 mm) compared with the Rotterdam Study (1 mm), which may have influenced the AI scores. However, the maintained high performance across both distinct cohorts suggests that the impact of domain shift was limited and did not substantially compromise system robustness. Future investigation stratified by acquisition parameters would better assess performance stability. Fourth, the biological basis of the association between AI scores and TBI remains unclear. Visualization techniques such as Grad-CAM would be necessary to identify image features prioritized by the AI system and to clarify the interpretability of AI predictions and their consistency with radiologists’ diagnostic inference. Future research incorporating such methods would enhance our understanding of the system’s decision-making process. Fifth, this study adopted the Fleischner Society definition of ILA [1]. In 2025, the American Thoracic Society (ATS) published a clinical statement on ILA, which provides updated diagnostic criteria and classification methods [32]. Future studies should validate this AI system using the latest ATS criteria to ensure consistency with current standards.

In conclusion, this automated ILA method showed robust performance for ILA detection across two unique population-based cohorts. The system demonstrated repeatability for identical CT images, and the AI score suggests associations with traction bronchiectasis/bronchiolectasis severity.

## Supplementary Information

Below is the link to the electronic supplementary material.

Supplementary file1 (DOCX 986 kb)

## Data Availability

Data generated or analyzed during the study are available from the corresponding author by request.
