# Supplementary material for: Automated interstitial lung abnormalities detection at CT: external validation and potential recognition of traction bronchiectasis/bronchiolectasis
Source: Jpn J Radiol. 2025 Dec 11;44(4):660–72. doi: 10.1007/s11604-025-01917-z (PMC13038644; doi:10.1007/s11604-025-01917-z)
Supplement: Supplementary file 1 — Supplementary file1 (DOCX 986 kb) [file 11604_2025_1917_MOESM1_ESM.docx]

**Supplementary materials**

**Table S1** Performance Measures of All 12 Models in the Rotterdam Study

| Section Inference Method, Case Inference Method, and Case Inference Classifier | AUC | Threshold = 0.5 | | Threshold = Youden Index | | Youden Index |
| --- | --- | --- | --- | --- | --- | --- |
|  |  | Sensitivity | Specificity | Sensitivity | Specificity |  |
| Two-label section inference |  |  |  |  |  |  |
| Two-label case inference |  |  |  |  |  |  |
| SVM | 0.8406 (0.804, 0.877) | 0.434 (0.343, 0.535); 43/99 | 0.927 (0.909, 0.944); 772/833 | 0.909 (0.737, 0.960); 90/99 | 0.637 (0.600, 0.828); 531/833 | 0.159 |
| RF | 0.8414 (0.804, 0.879) | 0.455 (0.354, 0.545); 45/99 | 0.928 (0.910, 0.945); 773/833 | 0.909 (0.778, 0.960); 90/99 | 0.649 (0.615, 0.791); 541/833 | 0.098 |
| CNN | 0.750 (0.693, 0.808) | 0.495 (0.394, 0.596); 49/99 | 0.866 (0.842, 0.887); 721/833 | 0.838 (0.717, 0.899); 83/99 | 0.672 (0.640, 0.789); 560/833 | < 0.001 |
| Three-label case inference |  |  |  |  |  |  |
| SVM | 0.835 (0.794, 0.876) | 0.768 (0.677, 0.848); 76/99 | 0.754 (0.723, 0.784); 628/833 | 0.889 (0.707, 0.949); 88/99 | 0.664 (0.629, 0.849); 553/833 | 0.344 |
| RF | 0.835 (0.799, 0.870) | 0.525 (0.424, 0.626); 52/99 | 0.870 (0.846, 0.892); 725/833 | 0.838 (0.778, 0.960); 83/99 | 0.700 (0.587, 0.778); 583/833 | 0.276 |
| CNN | 0.624 (0.549, 0.698) | 0.485 (0.384, 0.586); 48/99 | 0.818 (0.790, 0.844); 681/833 | 0.505 (0.364, 0.636); 50/99 | 0.810 (0.725, 0.910); 675/833 | 0.488 |
| Three-label section inference |  |  |  |  |  |  |
| Two-label case inference |  |  |  |  |  |  |
| SVM | 0.836 (0.801, 0.872) | 0.283 (0.202, 0.374); 28/99 | 0.970 (0.958, 0.981); 808/833 | 0.818 (0.768, 0.950); 81/99 | 0.726 (0.564, 0.765); 605/833 | 0.130 |
| RF | 0.797 (0.747, 0.846) | 0.263 (0.182, 0.343); 26/99 | 0.969 (0.956, 0.980); 807/833 | 0.798 (0.707, 0.889); 79/99 | 0.713 (0.663, 0.784); 594/833 | 0.046 |
| CNN | 0.774 (0.717, 0.832) | 0.212 (0.141, 0.293); 21/99 | 0.971 (0.959, 0.982); 809/833 | 0.758 (0.677, 0.859); 75/99 | 0.761 (0.676, 0.807); 634/833 | < 0.001 |
| Three-label case inference |  |  |  |  |  |  |
| SVM | 0.838 (0.802, 0.874) | 0.283 (0.192, 0.374); 28/99 | 0.960 (0.947, 0.972); 800/833 | 0.828 (0.768, 0.929); 82/99 | 0.731 (0.651, 0.778); 609/833 | 0.169 |
| RF | 0.825 (0.784, 0.865) | 0.465 (0.364, 0.566); 46/99 | 0.897 (0.875, 0.917); 747/833 | 0.788 (0.677, 0.960); 78/99 | 0.709 (0.541, 0.832); 591/833 | 0.213 |
| CNN | 0.753 (0.693, 0.813) | 0.364 (0.273, 0.465); 36/99 | 0.945 (0.928, 0.960); 787/833 | 0.727 (0.535, 0.869); 72/99 | 0.705 (0.585, 0.882); 587/833 | < 0.001 |

Data in parentheses are 95% CIs. Proportions are numbers of participants. *AUC* area under the receiver operating characteristic curve, *CNN* convolutional neural network, *RF* random forest, *SVM* support vector machine

**Table S2** Performance Measures of All 12 Models in the AGES-Reykjavik Study

| Section Inference Method, Case Inference Method, and Case Inference Classifier | AUC | Threshold = 0.5 | | Threshold = Youden Index | | Youden Index |
| --- | --- | --- | --- | --- | --- | --- |
|  |  | Sensitivity | Specificity | Sensitivity | Specificity |  |
| Two-label section inference |  |  |  |  |  |  |
| Two-label case inference |  |  |  |  |  |  |
| SVM | 0.810 (0.785, 0.835) | 0.520 (0.470, 0.565); 196/377 | 0.909 (0.901, 0.917); 4423/4865 | 0.671 (0.599, 0.769); 253/377 | 0.818 (0.736, 0.881); 3982/4865 | 0.340 |
| RF | 0.813 (0.788, 0.838) | 0.549 (0.501, 0.599); 207/377 | 0.902 (0.894, 0.911); 4388/4865 | 0.698 (0.629, 0.775); 263/377 | 0.795 (0.733, 0.847); 3868/4865 | 0.283 |
| CNN | 0.750 (0.721, 0.778) | 0.538 (0.488, 0.589); 203/377 | 0.851 (0.840, 0.861); 4138/4865 | 0.716 (0.634, 0.820); 270/377 | 0.724 (0.637, 0.794); 3521/4865 | < 0.001 |
| Three-label case inference |  |  |  |  |  |  |
| SVM | 0.809 (0.784, 0.835) | 0.764 (0.721, 0.804); 288/377 | 0.717 (0.705, 0.729); 3487/4865 | 0.735 (0.653, 0.785); 277/377 | 0.771 (0.737, 0.832); 3752/4865 | 0.573 |
| RF | 0.811 (0.786, 0.836) | 0.631 (0.581, 0.676); 238/377 | 0.849 (0.838, 0.859); 4129/4865 | 0.637 (0.602, 0.830); 240/377 | 0.846 (0.662, 0.875); 4116/4865 | 0.496 |
| CNN | 0.668 (0.635, 0.701) | 0.504 (0.454, 0.557); 190/377 | 0.770 (0.759, 0.782); 3748/4865 | 0.599 (0.406, 0.687); 226/377 | 0.692 (0.609, 0.882); 3367/4865 | 0.317 |
| Three-label section inference |  |  |  |  |  |  |
| Two-label case inference |  |  |  |  |  |  |
| SVM | 0.815 (0.790, 0.840) | 0.448 (0.401, 0.496); 169/377 | 0.948 (0.942, 0.954); 4613/4865 | 0.653 (0.605, 0.732); 246/377 | 0.846 (0.780, 0.882); 4114/4865 | 0.290 |
| RF | 0.810 (0.784, 0.836) | 0.454 (0.406, 0.501); 171/377 | 0.950 (0.944, 0.956); 4623/4865 | 0.621 (0.586, 0.764); 234/377 | 0.870 (0.735, 0.880); 4232/4865 | 0.257 |
| CNN | 0.789 (0.760, 0.818) | 0.382 (0.332, 0.430); 144/377 | 0.958 (0.952, 0.963); 4659/4865 | 0.735 (0.597, 0.777); 277/377 | 0.752 (0.724, 0.878); 3659/4865 | < 0.001 |
| Three-label case inference |  |  |  |  |  |  |
| SVM | 0.823 (0.798, 0.847) | 0.525 (0.475, 0.576); 198/377 | 0.927 (0.920, 0.935); 4512/4865 | 0.687 (0.637, 0.780); 259/377 | 0.826 (0.739, 0.864); 4018/4865 | 0.329 |
| RF | 0.813 (0.787, 0.838) | 0.642 (0.592, 0.687); 242/377 | 0.840 (0.830, 0.851); 4089/4865 | 0.687 (0.586, 0.769); 259/377 | 0.801 (0.715, 0.888); 3897/4865 | 0.438 |
| CNN | 0.812 (0.788, 0.837) | 0.570 (0.523, 0.621) | 0.895 (0.886, 0.903) | 0.719 (0.578, 0.812) | 0.760 (0.669, 0.899) | 0.002 |

Data in parentheses are 95% CIs. Proportions are numbers of participants. *AGES-Reykjavik Study* Age Gene/Environment Susceptibility Reykjavik Study, *AUC* area under the receiver operating characteristic curve, *CNN* convolutional neural network, *RF* random forest, *SVM* support vector machine

**Table S3 Performance Measures of Optimal Models in Sensitivity Analysis Excluding Indeterminate-ILA Cases**

| Study | Section Inference Method, Case Inference Method, and Case Inference Classifier | AUC | Threshold = 0.5 | | Threshold = Youden Index | | Youden Index |
| --- | --- | --- | --- | --- | --- | --- | --- |
|  |  |  | Sensitivity | Specificity | Sensitivity | Specificity |  |
| Rotterdam Study | Two-label section inference, Two-label case inference, and RF classifier | 0.894 (0.861, 0.927) | 0.455 (0.364, 0.545); 45/99 | 0.969 (0.953, 0.984); 498/514 | 0.919 (0.838, 0.970); 91/99 | 0.759 (0.720, 0.833); 390/514 | 0.092 |
| AGES-Reykjavik Study | Three-label section inference, Three-label case inference, and SVM classifier | 0.864 (0.841, 0.886) | 0.525 (0.472, 0.576); 198/377 | 0.974 (0.969, 0.980); 3116/3198 | 0.687 (0.639, 0.732); 259/377 | 0.898 (0.888, 0.909); 2873/3198 | 0.329 |

Data in parentheses are 95% CIs. Proportions are numbers of participants

*AGES-Reykjavik Study* Age Gene/Environment Susceptibility Reykjavik Study, *AUC* area under the receiver operating characteristic curve, *ILA* interstitial lung abnormalities, *RF* random forest, *SVM* support vector machine

**Table S4** *p* Values of the Two Readers vs. AI score Analysis in the Rotterdam Study

| Two readers result^*^ | *p* Value | | | | |
| --- | --- | --- | --- | --- | --- |
|  | vs. 0/1 | vs. 0/2 | vs. 1/1 | vs. 1/2 | vs. 2/2 |
| 0/0 | *p* = 0.006 | *p* = 0.01 | *p* < 0.001 | *p* < 0.001 | *p* < 0.001 |
| 0/1 | - | *p* = 0.22 | *p* = 0.001 | *p* < 0.001 | *p* < 0.001 |
| 0/2 |  | - | *p* = 0.19 | *p* = 0.006 | *p* < 0.001 |
| 1/1 |  |  | - | *p* = 0.002 | *p* < 0.001 |
| 1/2 |  |  |  | - | *p* < 0.001 |
| 2/2 |  |  |  |  | - |

*AI* artificial intelligence, *ILA* interstitial lung abnormalities

^*^ 0, 1, and 2 represent No-ILA, Indeterminate-ILA, and ILA, respectively. 0/0, 1/1, and 2/2 indicate the cases unanimously classified as No-ILA, Indeterminate-ILA, and ILA by both readers, respectively. While 0/1, 0/2, and 1/2 indicate the cases with reader disagreement, for instance, 0/1 indicates one reader classified it as No-ILA while the other as Indeterminate-ILA

**Table S5** *p* Values of the Two Readers vs. AI score Analysis in the AGES-Reykjavik Study

| Two readers result^*^ | *p* Value | | | | |
| --- | --- | --- | --- | --- | --- |
|  | vs. 0/1 | vs. 0/2 | vs. 1/1 | vs. 1/2 | vs. 2/2 |
| 0/0 | *p* < 0.001 | *p* = 0.004 | *p* < 0.001 | *p* < 0.001 | *p* < 0.001 |
| 0/1 | - | *p* = 0.32 | *p* < 0.001 | *p* < 0.001 | *p* < 0.001 |
| 0/2 |  | - | *p* = 0.002 | *p* < 0.001 | *p* < 0.001 |
| 1/1 |  |  | - | *p* < 0.001 | *p* < 0.001 |
| 1/2 |  |  |  | - | *p* < 0.001 |
| 2/2 |  |  |  |  | - |

*AI* artificial intelligence, *AGES-Reykjavik Study* Age Gene/Environment Susceptibility Reykjavik Study, *ILA* interstitial lung abnormalities

^*^ 0, 1, and 2 represent No-ILA, Indeterminate-ILA, and ILA, respectively. 0/0, 1/1, and 2/2 indicate the cases unanimously classified as No-ILA, Indeterminate-ILA, and ILA by both readers, respectively. While 0/1, 0/2, and 1/2 indicate the cases with reader disagreement, for instance, 0/1 indicates one reader classified it as No-ILA while the other as Indeterminate-ILA

**Table S6** All results of analyzing 30 randomly selected cases three times in 12 models

| Case No. | 3-3-SVM 1st | 3-3-SVM 2nd | 3-3-SVM 3rd | 3-3-RF 1st | 3-3-RF 2nd | 3-3-RF 3rd |
| --- | --- | --- | --- | --- | --- | --- |
| 1 | 0.130241 | 0.130241 | 0.130241 | 0.225 | 0.225 | 0.225 |
| 2 | 0.130578 | 0.130578 | 0.130578 | 0.2 | 0.2 | 0.2 |
| 3 | 0.099623 | 0.099623 | 0.099623 | 0.025 | 0.025 | 0.025 |
| 4 | 0.100684 | 0.100684 | 0.100684 | 0.1 | 0.1 | 0.1 |
| 5 | 0.099916 | 0.099916 | 0.099916 | 0 | 0 | 0 |
| 6 | 0.101655 | 0.101655 | 0.101655 | 0 | 0 | 0 |
| 7 | 0.361969 | 0.361969 | 0.361969 | 0.3 | 0.3 | 0.3 |
| 8 | 0.100226 | 0.100226 | 0.100226 | 0.075 | 0.075 | 0.075 |
| 9 | 0.307506 | 0.307506 | 0.307506 | 0.45 | 0.45 | 0.45 |
| 10 | 0.111465 | 0.111465 | 0.111465 | 0.175 | 0.175 | 0.175 |
| 11 | 0.099524 | 0.099524 | 0.099524 | 0.025 | 0.025 | 0.025 |
| 12 | 0.128769 | 0.128769 | 0.128769 | 0.175 | 0.175 | 0.175 |
| 13 | 0.120369 | 0.120369 | 0.120369 | 0 | 0 | 0 |
| 14 | 0.102886 | 0.102886 | 0.102886 | 0.025 | 0.025 | 0.025 |
| 15 | 0.103101 | 0.103101 | 0.103101 | 0.2 | 0.2 | 0.2 |
| 16 | 0.226293 | 0.226293 | 0.226293 | 0.35 | 0.35 | 0.35 |
| 17 | 0.102679 | 0.102679 | 0.102679 | 0.125 | 0.125 | 0.125 |
| 18 | 0.129522 | 0.129522 | 0.129522 | 0 | 0 | 0 |
| 19 | 0.100949 | 0.100949 | 0.100949 | 0.05 | 0.05 | 0.05 |
| 20 | 0.23061 | 0.23061 | 0.23061 | 0.4 | 0.4 | 0.4 |
| 21 | 0.260792 | 0.260792 | 0.260792 | 0.425 | 0.425 | 0.425 |
| 22 | 0.138549 | 0.138549 | 0.138549 | 0.3 | 0.3 | 0.3 |
| 23 | 0.206989 | 0.206989 | 0.206989 | 0.425 | 0.425 | 0.425 |
| 24 | 0.190961 | 0.190961 | 0.190961 | 0.3 | 0.3 | 0.3 |
| 25 | 0.970156 | 0.970156 | 0.970156 | 1 | 1 | 1 |
| 26 | 0.24077 | 0.24077 | 0.24077 | 0.25 | 0.25 | 0.25 |
| 27 | 0.174096 | 0.174096 | 0.174096 | 0.175 | 0.175 | 0.175 |
| 28 | 0.793239 | 0.793239 | 0.793239 | 0.975 | 0.975 | 0.975 |
| 29 | 0.121193 | 0.121193 | 0.121193 | 0.1 | 0.1 | 0.1 |
| 30 | 0.106218 | 0.106218 | 0.106218 | 0.075 | 0.075 | 0.075 |

| Case No. | 3-3-CNN 1st | 3-3-CNN 2nd | 3-3-CNN 3rd | 3-2-SVM 1st | 3-2-SVM 2nd | 3-2-SVM 3rd |
| --- | --- | --- | --- | --- | --- | --- |
| 1 | 0.000114 | 0.000114 | 0.000114 | 0.093901 | 0.093901 | 0.093901 |
| 2 | 0.000049 | 0.000049 | 0.000049 | 0.089248 | 0.089248 | 0.089248 |
| 3 | 0.000013 | 0.000013 | 0.000013 | 0.071406 | 0.071406 | 0.071406 |
| 4 | 0.000014 | 0.000014 | 0.000014 | 0.071898 | 0.071898 | 0.071898 |
| 5 | 0.000013 | 0.000013 | 0.000013 | 0.071773 | 0.071773 | 0.071773 |
| 6 | 0.000014 | 0.000014 | 0.000014 | 0.073525 | 0.073525 | 0.073525 |
| 7 | 0.375936 | 0.375936 | 0.375936 | 0.314223 | 0.314223 | 0.314223 |
| 8 | 0.000014 | 0.000014 | 0.000014 | 0.072013 | 0.072013 | 0.072013 |
| 9 | 0.002603 | 0.002603 | 0.002603 | 0.273703 | 0.273703 | 0.273703 |
| 10 | 0.000015 | 0.000015 | 0.000015 | 0.079053 | 0.079053 | 0.079053 |
| 11 | 0.000013 | 0.000013 | 0.000013 | 0.071342 | 0.071342 | 0.071342 |
| 12 | 0.000014 | 0.000014 | 0.000014 | 0.090731 | 0.090731 | 0.090731 |
| 13 | 0.000013 | 0.000013 | 0.000013 | 0.098885 | 0.098885 | 0.098885 |
| 14 | 0.000015 | 0.000015 | 0.000015 | 0.074252 | 0.074252 | 0.074252 |
| 15 | 0.000013 | 0.000013 | 0.000013 | 0.074331 | 0.074331 | 0.074331 |
| 16 | 0.000079 | 0.000079 | 0.000079 | 0.185759 | 0.185759 | 0.185759 |
| 17 | 0.000015 | 0.000015 | 0.000015 | 0.072806 | 0.072806 | 0.072806 |
| 18 | 0.000013 | 0.000013 | 0.000013 | 0.09981 | 0.09981 | 0.09981 |
| 19 | 0.000014 | 0.000014 | 0.000014 | 0.072277 | 0.072277 | 0.072277 |
| 20 | 0.001779 | 0.001779 | 0.001779 | 0.168465 | 0.168465 | 0.168465 |
| 21 | 0.029636 | 0.029636 | 0.029636 | 0.208982 | 0.208982 | 0.208982 |
| 22 | 0.000097 | 0.000097 | 0.000097 | 0.065944 | 0.065944 | 0.065944 |
| 23 | 0.001015 | 0.001015 | 0.001015 | 0.159364 | 0.159364 | **0.159365** |
| 24 | 0.001162 | 0.001162 | 0.001162 | 0.153365 | 0.153365 | 0.153365 |
| 25 | 1 | 1 | 1 | 0.967501 | 0.967501 | 0.967501 |
| 26 | 0.000292 | 0.000292 | 0.000292 | 0.191224 | 0.191224 | 0.191224 |
| 27 | 0.000013 | 0.000013 | 0.000013 | 0.136947 | 0.136947 | 0.136947 |
| 28 | 0.999548 | 0.999548 | 0.999548 | 0.766401 | 0.766401 | 0.766401 |
| 29 | 0.000022 | 0.000022 | 0.000022 | 0.086746 | 0.086746 | 0.086746 |
| 30 | 0.000016 | 0.000016 | 0.000016 | 0.076963 | 0.076963 | 0.076963 |

| Case No. | 3-2-RF 1st | 3-2-RF 2nd | 3-2-RF 3rd | 3-2-CNN 1st | 3-2-CNN 2nd | 3-2-CNN 3rd |
| --- | --- | --- | --- | --- | --- | --- |
| 1 | 0.020768 | 0.020768 | 0.020768 | 0 | 0 | 0 |
| 2 | 0.030511 | 0.030511 | 0.030511 | 0 | 0 | 0 |
| 3 | 0.020768 | 0.020768 | 0.020768 | 0 | 0 | 0 |
| 4 | 0.020768 | 0.020768 | 0.020768 | 0 | 0 | 0 |
| 5 | 0.020768 | 0.020768 | 0.020768 | 0 | 0 | 0 |
| 6 | 0.020768 | 0.020768 | 0.020768 | 0 | 0 | 0 |
| 7 | 0.229215 | 0.229215 | 0.229215 | 0.899085 | 0.899085 | 0.899085 |
| 8 | 0.020768 | 0.020768 | 0.020768 | 0 | 0 | 0 |
| 9 | 0.239419 | 0.239419 | 0.239419 | 0 | 0 | 0 |
| 10 | 0.020768 | 0.020768 | 0.020768 | 0 | 0 | 0 |
| 11 | 0.020768 | 0.020768 | 0.020768 | 0 | 0 | 0 |
| 12 | 0.020768 | 0.020768 | 0.020768 | 0 | 0 | 0 |
| 13 | 0.033627 | 0.033627 | 0.033627 | 0 | 0 | 0 |
| 14 | 0.020768 | 0.020768 | 0.020768 | 0 | 0 | 0 |
| 15 | 0.020768 | 0.020768 | 0.020768 | 0 | 0 | 0 |
| 16 | 0.13181 | 0.13181 | 0.13181 | 0 | 0 | 0 |
| 17 | 0.020768 | 0.020768 | 0.020768 | 0 | 0 | 0 |
| 18 | 0.020768 | 0.020768 | 0.020768 | 0 | 0 | 0 |
| 19 | 0.030511 | 0.030511 | 0.030511 | 0 | 0 | 0 |
| 20 | 0.053398 | 0.053398 | 0.053398 | 0 | 0 | 0 |
| 21 | 0.023625 | 0.023625 | 0.023625 | 0 | 0 | 0 |
| 22 | 0.020768 | 0.020768 | 0.020768 | 0 | 0 | 0 |
| 23 | 0.020768 | 0.020768 | 0.020768 | 0 | 0 | 0 |
| 24 | 0.062293 | 0.062293 | 0.062293 | 0 | 0 | 0 |
| 25 | 0.998518 | 0.998518 | 0.998518 | 1 | 1 | 1 |
| 26 | 0.037434 | 0.037434 | 0.037434 | 0 | 0 | 0 |
| 27 | 0.064638 | 0.064638 | 0.064638 | 0 | 0 | 0 |
| 28 | 0.728397 | 0.728397 | 0.728397 | 0.736324 | 0.736324 | **0.736328** |
| 29 | 0.030511 | 0.030511 | 0.030511 | 0 | 0 | 0 |
| 30 | 0.020768 | 0.020768 | 0.020768 | 0 | 0 | 0 |

| Case No. | 2-3-SVM 1st | 2-3-SVM 2nd | 2-3-SVM 3rd | 2-3-RF 1st | 2-3-RF 2nd | 2-3-RF 3rd |
| --- | --- | --- | --- | --- | --- | --- |
| 1 | 0.098766 | 0.098766 | 0.098766 | 0.191079 | 0.191079 | 0.191079 |
| 2 | 0.201427 | 0.201427 | 0.201427 | 0.271813 | 0.271813 | 0.271813 |
| 3 | 0.099771 | 0.099771 | 0.099771 | 0.150062 | 0.150062 | 0.150062 |
| 4 | 0.280684 | 0.280684 | 0.280684 | 0.150062 | 0.150062 | 0.150062 |
| 5 | 0.18062 | 0.18062 | 0.18062 | 0.150062 | 0.150062 | 0.150062 |
| 6 | 0.133497 | 0.133497 | **0.133496** | 0.150062 | 0.150062 | 0.150062 |
| 7 | 0.546087 | 0.546087 | 0.546087 | 0.331965 | 0.331965 | 0.331965 |
| 8 | 0.082184 | 0.082184 | 0.082184 | 0.168031 | 0.168031 | 0.168031 |
| 9 | 0.801452 | 0.801452 | 0.801452 | 0.709051 | 0.709051 | 0.709051 |
| 10 | 0.176444 | 0.176444 | 0.176444 | 0.150062 | 0.150062 | 0.150062 |
| 11 | 0.097312 | 0.097312 | 0.097312 | 0.168031 | 0.168031 | 0.168031 |
| 12 | 0.099662 | 0.099662 | 0.099662 | 0.150062 | 0.150062 | 0.150062 |
| 13 | 0.098928 | 0.098928 | 0.098928 | 0.150062 | 0.150062 | 0.150062 |
| 14 | 0.099055 | 0.099055 | 0.099055 | 0.150062 | 0.150062 | 0.150062 |
| 15 | 0.100567 | 0.100567 | 0.100567 | 0.150062 | 0.150062 | 0.150062 |
| 16 | 0.099409 | 0.099409 | 0.099409 | 0.172032 | 0.172032 | 0.172032 |
| 17 | 0.095112 | 0.095112 | 0.095112 | 0.150062 | 0.150062 | 0.150062 |
| 18 | 0.092835 | 0.092835 | 0.092835 | 0.150062 | 0.150062 | 0.150062 |
| 19 | 0.099771 | 0.099771 | 0.099771 | 0.168031 | 0.168031 | 0.168031 |
| 20 | 0.633144 | 0.633144 | 0.633144 | 0.477556 | 0.477556 | 0.477556 |
| 21 | 0.756641 | 0.756641 | 0.756641 | 0.468008 | 0.468008 | 0.468008 |
| 22 | 0.301801 | 0.301801 | **0.301800** | 0.253187 | 0.253187 | 0.253187 |
| 23 | 0.53648 | 0.53648 | 0.53648 | 0.359346 | 0.359346 | 0.359346 |
| 24 | **0.625375** | 0.625376 | 0.625376 | 0.439387 | 0.439387 | 0.439387 |
| 25 | 0.856527 | 0.856527 | 0.856527 | 0.832454 | 0.832454 | 0.832454 |
| 26 | 0.612388 | 0.612388 | **0.612387** | 0.455481 | 0.455481 | 0.455481 |
| 27 | 0.370404 | 0.370404 | 0.370404 | 0.275156 | 0.275156 | 0.275156 |
| 28 | 0.889566 | 0.889566 | 0.889566 | 0.943912 | 0.943912 | 0.943912 |
| 29 | 0.079055 | 0.079055 | 0.079055 | 0.150062 | 0.150062 | 0.150062 |
| 30 | 0.496334 | 0.496334 | **0.496335** | 0.431586 | 0.431586 | 0.431586 |

| Case No. | 2-3-CNN 1st | 2-3-CNN 2nd | 2-3-CNN 3rd | 2-2-SVM 1st | 2-2-SVM 2nd | 2-2-SVM 3rd |
| --- | --- | --- | --- | --- | --- | --- |
| 1 | 0.468691 | 0.468691 | 0.468691 | 0.08052 | 0.08052 | 0.08052 |
| 2 | 0.000118 | 0.000118 | 0.000118 | 0.122264 | 0.122264 | 0.122264 |
| 3 | 0.094069 | 0.094069 | 0.094069 | 0.078442 | 0.078442 | 0.078442 |
| 4 | 0.012774 | 0.012774 | 0.012774 | 0.09637 | 0.09637 | 0.09637 |
| 5 | 0.093788 | 0.093788 | 0.093788 | 0.11035 | 0.11035 | 0.11035 |
| 6 | 0.096124 | 0.096124 | 0.096124 | 0.09404 | 0.09404 | 0.09404 |
| 7 | 0.032543 | 0.032543 | 0.032543 | 0.300609 | 0.300609 | 0.300609 |
| 8 | 0.088988 | 0.088988 | 0.088988 | 0.079618 | 0.079618 | 0.079618 |
| 9 | 0.97341 | 0.97341 | 0.97341 | 0.605273 | 0.605273 | 0.605273 |
| 10 | 0.010261 | 0.010261 | 0.010261 | 0.084065 | 0.084065 | 0.084065 |
| 11 | 0.090071 | 0.090071 | 0.090071 | 0.078874 | 0.078874 | 0.078874 |
| 12 | 0.09162 | 0.09162 | 0.09162 | 0.078548 | 0.078548 | 0.078548 |
| 13 | 0.09501 | 0.09501 | 0.09501 | 0.078604 | 0.078604 | 0.078604 |
| 14 | 0.09962 | 0.09962 | 0.09962 | 0.078786 | 0.078786 | 0.078786 |
| 15 | 0.970595 | 0.970595 | 0.970595 | 0.080236 | 0.080236 | 0.080236 |
| 16 | 0.071653 | 0.071653 | 0.071653 | 0.079883 | 0.079883 | 0.079883 |
| 17 | 0.092928 | 0.092928 | 0.092928 | 0.078314 | 0.078314 | 0.078314 |
| 18 | 0.098214 | 0.098214 | 0.098214 | 0.081212 | 0.081212 | 0.081212 |
| 19 | 0.09659 | 0.09659 | 0.09659 | 0.078513 | 0.078513 | 0.078513 |
| 20 | **0.737848** | 0.737849 | 0.737849 | 0.367413 | 0.367413 | 0.367413 |
| 21 | 0.97849 | 0.97849 | 0.97849 | 0.490516 | 0.490516 | 0.490516 |
| 22 | 0.002369 | 0.002369 | 0.002369 | 0.099755 | 0.099755 | 0.099755 |
| 23 | 0.126612 | 0.126612 | **0.12661** | 0.199772 | 0.199772 | 0.199772 |
| 24 | 0.825774 | 0.825774 | 0.825774 | 0.299319 | 0.299319 | 0.299319 |
| 25 | 0.894179 | 0.894179 | 0.894179 | 0.830112 | 0.830112 | 0.830112 |
| 26 | 0.086371 | 0.086371 | **0.086373** | 0.392539 | 0.392539 | 0.392539 |
| 27 | 0.03323 | 0.03323 | 0.03323 | 0.134298 | 0.134298 | 0.134298 |
| 28 | 0.882565 | 0.882565 | 0.882565 | 0.796911 | 0.796911 | **0.796912** |
| 29 | 0.231174 | 0.231174 | 0.231174 | 0.082935 | 0.082935 | 0.082935 |
| 30 | 0.305856 | 0.305856 | **0.305857** | 0.231677 | 0.231677 | 0.231677 |

| Case No. | 2-2-RF 1st | 2-2-RF 2nd | 2-2-RF 3rd | 2-2-CNN 1st | 2-2-CNN 2nd | 2-2-CNN 3rd |
| --- | --- | --- | --- | --- | --- | --- |
| 1 | 0.057644 | 0.057644 | 0.057644 | 0.000001 | 0.000001 | 0.000001 |
| 2 | 0.138736 | 0.138736 | 0.138736 | 0.003479 | 0.003479 | 0.003479 |
| 3 | 0.013056 | 0.013056 | 0.013056 | 0.000001 | 0.000001 | 0.000001 |
| 4 | 0.020866 | 0.020866 | 0.020866 | 0.000001 | 0.000001 | 0.000001 |
| 5 | 0.013056 | 0.013056 | 0.013056 | 0.000001 | 0.000001 | 0.000001 |
| 6 | 0.013056 | 0.013056 | 0.013056 | 0.000001 | 0.000001 | 0.000001 |
| 7 | 0.200428 | 0.200428 | 0.200428 | 0.000009 | 0.000009 | 0.000009 |
| 8 | 0.013056 | 0.013056 | 0.013056 | 0.000001 | 0.000001 | 0.000001 |
| 9 | 0.626906 | 0.626906 | 0.626906 | 1 | 1 | 1 |
| 10 | 0.020437 | 0.020437 | 0.020437 | 0.000001 | 0.000001 | 0.000001 |
| 11 | 0.019723 | 0.019723 | 0.019723 | 0.000001 | 0.000001 | 0.000001 |
| 12 | 0.022306 | 0.022306 | 0.022306 | 0.000001 | 0.000001 | 0.000001 |
| 13 | 0.022878 | 0.022878 | 0.022878 | 0.000001 | 0.000001 | 0.000001 |
| 14 | 0.03192 | 0.03192 | 0.03192 | 0.000001 | 0.000001 | 0.000001 |
| 15 | 0.013056 | 0.013056 | 0.013056 | 0.000001 | 0.000001 | 0.000001 |
| 16 | 0.013473 | 0.013473 | 0.013473 | 0.000001 | 0.000001 | 0.000001 |
| 17 | 0.017524 | 0.017524 | 0.017524 | 0.000001 | 0.000001 | 0.000001 |
| 18 | 0.022306 | 0.022306 | 0.022306 | 0.000001 | 0.000001 | 0.000001 |
| 19 | 0.013056 | 0.013056 | 0.013056 | 0.000001 | 0.000001 | 0.000001 |
| 20 | 0.32887 | 0.32887 | 0.32887 | 0.007432 | 0.007432 | 0.007432 |
| 21 | 0.354841 | 0.354841 | 0.354841 | 0.000289 | 0.000289 | 0.000289 |
| 22 | 0.120475 | 0.120475 | 0.120475 | 0.000001 | 0.000001 | 0.000001 |
| 23 | 0.128109 | 0.128109 | 0.128109 | 0.008713 | 0.008713 | 0.008713 |
| 24 | 0.273075 | 0.273075 | 0.273075 | 0.012275 | 0.012275 | 0.012275 |
| 25 | 0.838904 | 0.838904 | 0.838904 | 0.99333 | 0.99333 | 0.99333 |
| 26 | 0.262721 | 0.262721 | 0.262721 | 0.996361 | 0.996361 | 0.996361 |
| 27 | 0.156496 | 0.156496 | 0.156496 | 0.000005 | 0.000005 | 0.000005 |
| 28 | 0.899201 | 0.899201 | 0.899201 | 0.999217 | 0.999217 | 0.999217 |
| 29 | 0.013681 | 0.013681 | 0.013681 | 0.000001 | 0.000001 | 0.000001 |
| 30 | 0.246349 | 0.246349 | 0.246349 | 0.999442 | 0.999442 | 0.999442 |

The model notation is represented as "section inference method-case inference method-case inference classifier." For instance, "3-3-SVM" represents a model with three-label section inference, three-label case inference, and SVM case inference classifier. Similarly, "2-3-CNN" refers to a model with two-label section inference, three-label case inference, and CNN case inference classifier. The values calculated by the AI system are shown in the table (truncated to six decimal places, with digits beyond the seventh decimal place discarded). Values that differed at the sixth decimal place are shown in **bold**. *CNN* convolution neural network, *RF* random forest, *SVM* support vector machine

**Fig. S1**


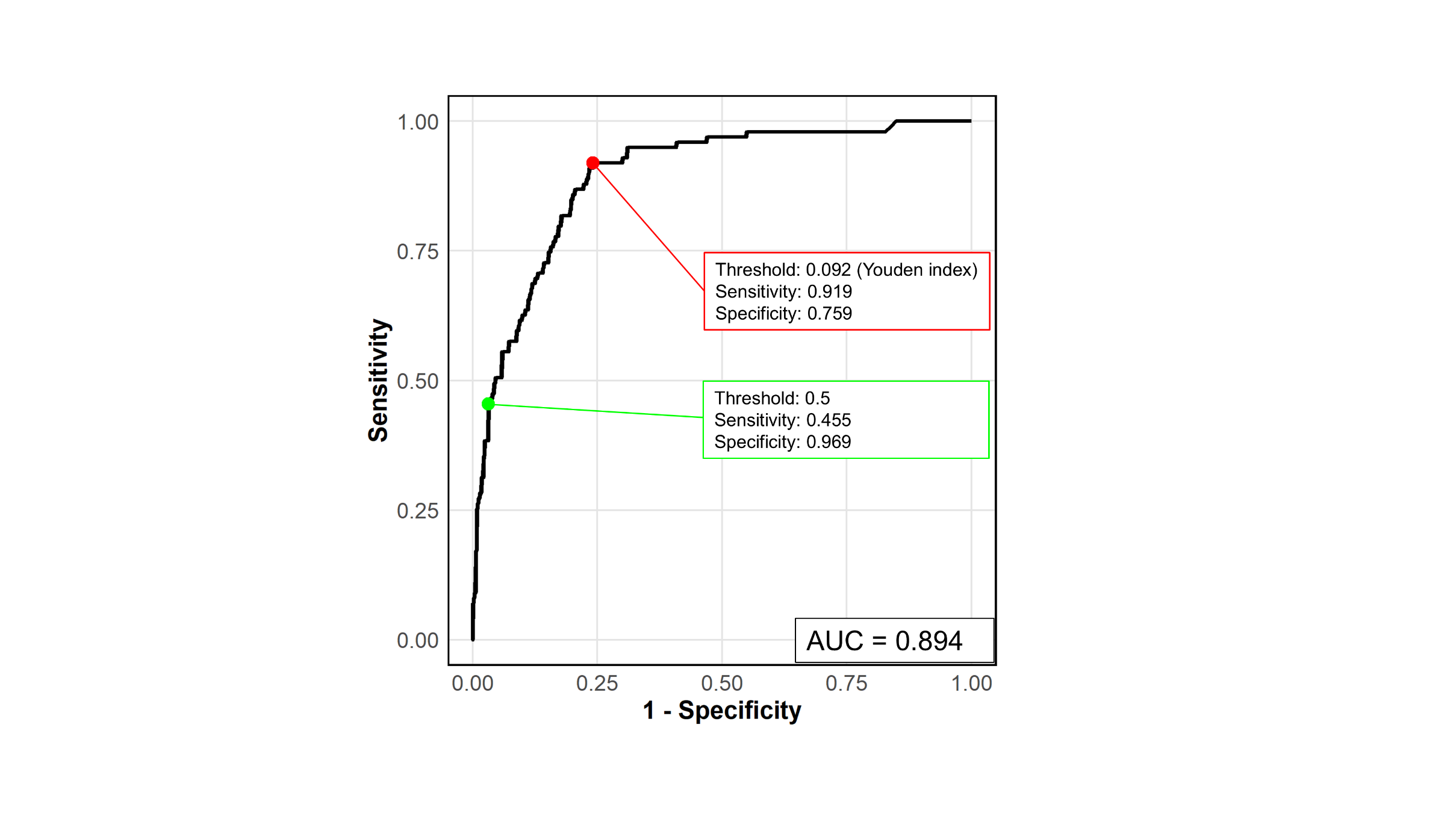


ROC curve for the optimal model in the sensitivity analysis excluding Indeterminate-ILA cases in the Rotterdam Study. The optimal model (two-label section inference, two-label case inference, and random forest classifier) achieved an AUC of 0.894. The sensitivity and specificity at a threshold of 0.5 and at the Youden index threshold are shown in the figure (green and red points, respectively). *AUC* area under the receiver operating characteristic curve, *ILA* interstitial lung abnormalities, *RF* random forest, *ROC* receiver operating characteristic

**Fig. S2**


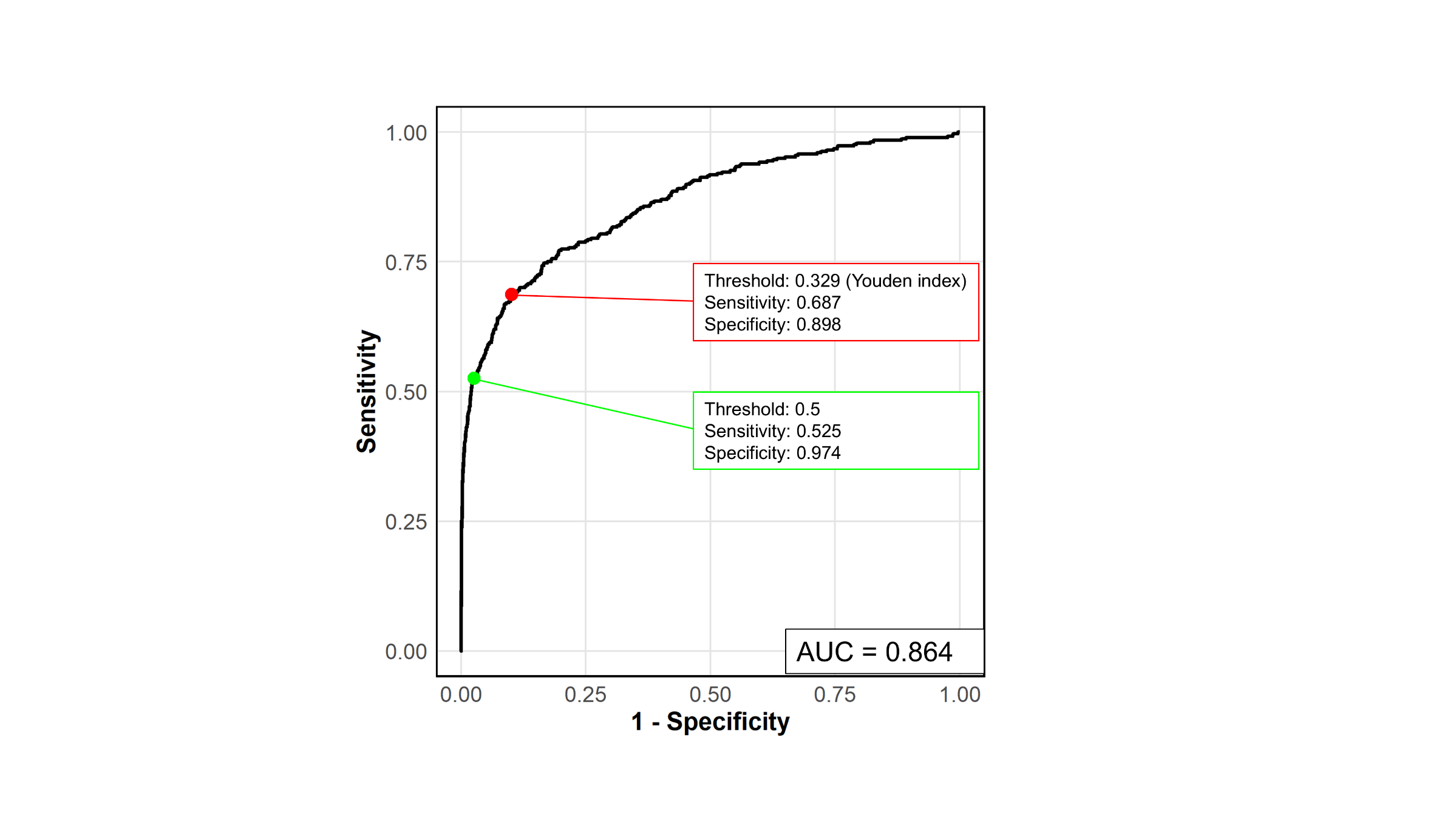


ROC curve for the optimal model in the sensitivity analysis excluding Indeterminate-ILA cases in the AGES-Reykjavik Study. The optimal model (three-label section inference, three-label case inference, and support vector machine) achieved an AUC of 0.864. The sensitivity and specificity at a threshold of 0.5 and at the Youden index threshold are shown in the figure (green and red points, respectively). *AGES-Reykjavik Study* Age Gene/Environment Susceptibility Reykjavik Study, *AUC* area under the receiver operating characteristic curve, *ILA* interstitial lung abnormalities, *ROC* receiver operating characteristic, *SVM* support vector machine

**Fig. S3**


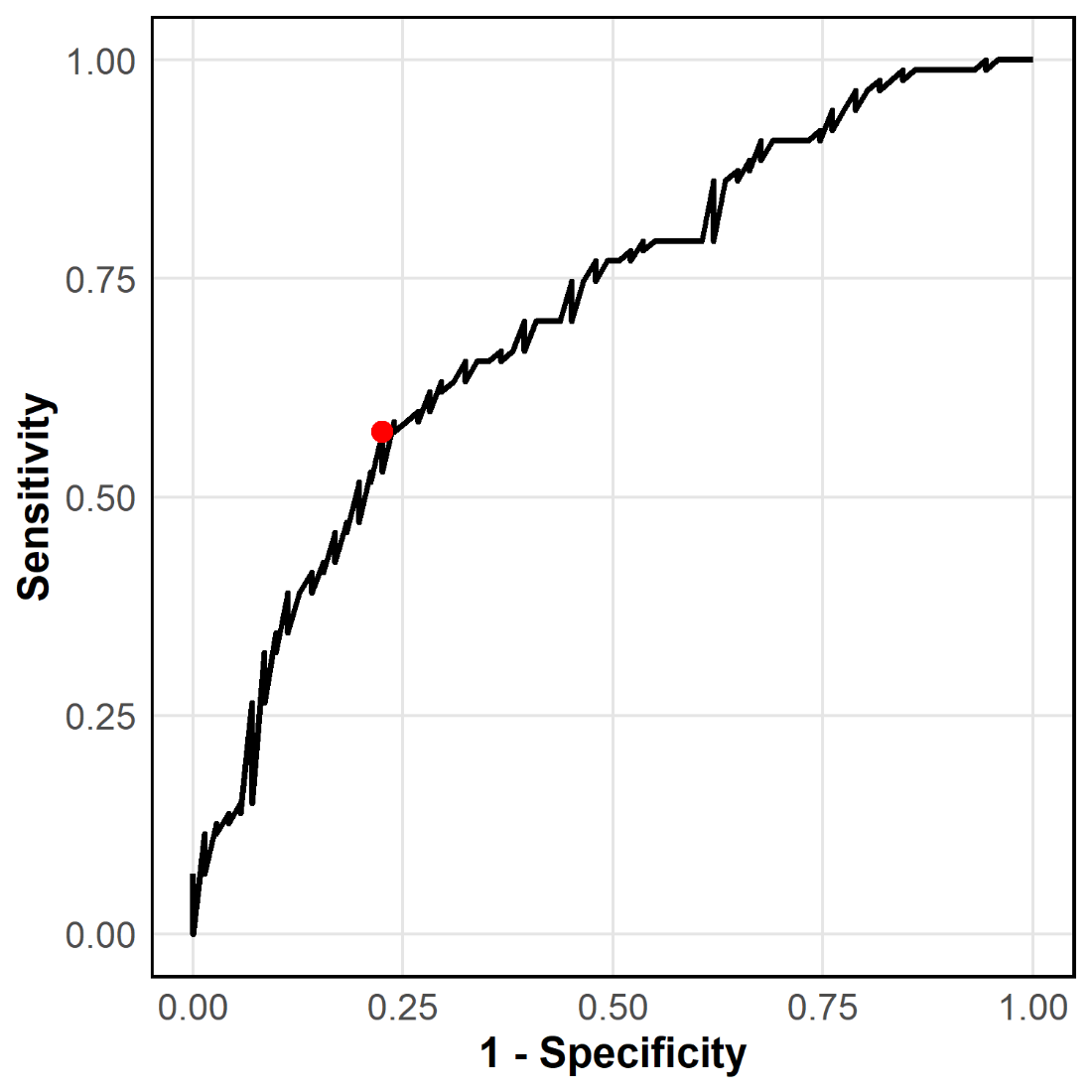


Receiver operating characteristic curve for ΔAI score discrimination of traction bronchiectasis/bronchiolectasis progression. The AUC was 0.710 (95% CI: 0.630, 0.791). The red point indicates the optimal threshold determined by the Youden index (threshold = 0.039), with sensitivity of 0.574 and specificity of 0.775. Positive predictive value was 0.758 and negative predictive value was 0.598. *AI* artificial intelligence, *AUC* area under the receiver operating characteristic curve
